# Network reconstruction from calcium imaging data of spontaneously bursting neuronal activity

**DOI:** 10.1186/1471-2202-14-S1-P139

**Published:** 2013-07-08

**Authors:** Olav Stetter, Javier Orlandi, Jordi Soriano, Demian Battaglia, Theo Geisel

**Affiliations:** 1 Max Planck Institute for Dynamics and Self-Organization, Göttingen, 37073, Germany; 2 Bernstein Center for Computational Neuroscience, Göttingen, 37073, Germany; 3 Universitat de Barcelona, Spain

## 

There is a growing interest in reconstructing the structural connectivity of a neuronal circuit based on observing its activity. This would allow for the study of bulk network properties, like the degree distribution or the clustering index, as well as facilitate a better understanding of neuronal function.

In particular, a causality measure called Transfer Entropy was proposed as a non-linear generalization of Granger Causality and applied to this question [1-3]. Studying signals of simulated calcium imaging, we point out the advantages of using a non-linear causality measure. It turns out however, that the causal influences are time-dependent despite the static nature of the simulated topology. This leads to the identification of a range of different dynamical regimes of the spontaneous activity in the network and, correspondingly, different effective connectivities. Interestingly, there exists one particular regime in which there is a very good overlap between the effective and structural (synaptic) connectivity, which can be captured using a new measure of causal interactions, the Generalized Transfer Entropy (GTE) [4]. We point out a number of features of the reconstructed networks using GTE, for instance the very good linear correlation of bulk network properties like the clustering coefficient, when comparing the reconstructed and physical networks. This points to, as is desirable, an unbiased reconstruction method.

We show how GTE can be applied to the detection of excitatory as well as of inhibitory connections, and compare with model-based approaches like cross-correlation or Bayesian inference methods [5]. The performance of the latter can break down due to the correlations in the network activity due to spontaneous bursting events.

Finally, we show how GTE can be applied to the analysis of real neurons and demonstrate the properties of network of dissociated, cultured neurons. We find a rich, non-random topology characterized by an elevated mean clustering coefficient and long-range connectivity profiles. Thus GTE is a promising method for the reconstruction of network connectivities, especially when taking into account its generality due to the model-free approach.
